# Dataset containing physiological amounts of spike-in proteins into murine C2C12 background as a ground truth quantitative LC-MS/MS reference

**DOI:** 10.1016/j.dib.2022.108435

**Published:** 2022-07-04

**Authors:** Julian Uszkoreit, Katalin Barkovits, Sandra Pacharra, Kathy Pfeiffer, Simone Steinbach, Katrin Marcus, Martin Eisenacher

**Affiliations:** a Medical Faculty, Medical Proteome Center^2^, Ruhr University Bochum, Bochum 44801, Germany; b Center for Protein Diagnostics (PRODI), Medical Proteome Analysis, Ruhr University Bochum, Bochum 44801, Germany; c Institute of Bio- and Geosciences (IBG-5) - Computational Metagenomics, Forschungszentrum Jülich GmbH, ELIXIR Germany, Jülich 52425, Germany; d ProtaGene GmbH, Dortmund 44227, Germany

**Keywords:** Proteomics, Mass spectrometry, Protein spike-in dataset, Complex proteomics standard, Quantitative ground truth dataset, C2C12 cell line

## Abstract

In this article, we present a data dependent acquisition (DDA) dataset which was generated as a reference and ground truth quantitative dataset. While initially used to compare samples measured with DDA and data independent acquisition (DIA) (Barkovits et al., 2020), the presented dataset holds potential value as a benchmark reference for any workflows working on DDA data. The entire dataset consists of 15 LC-MS/MS measurements composed of five distinct spike-in-states, each with three replicates. To generate the data set, a C2C12 (immortalized mouse myoblast) cell lysate was used as a complex background for five different states which were simulated by spiking 13 defined proteins at different concentrations. For this purpose, the cell lysate was used in a constant amount of 20 µg for all samples and different amounts of the 13 selected proteins ranging from 0.1  to 10 pmol were added, reflecting physiological amounts of proteins. Afterwards, all samples were tryptically digested using the same method. From each sample 200 ng tryptic peptides were measured in triplicates on a Q Exactive HF (Thermo Fisher Scientific). The mass range for MS1 was set to 350–1400 m/z with a resolution of 60,000 at 200 m/z. HCD fragmentation of the Top10 abundant precursor ions was performed at 27% NCE. The fragment analysis (MS2) was performed with a resolution of 30,000 at 200 m/z.

Additionally to the raw files, the dataset contains centroided mzML files and spectrum identification results for peptide identifications performed by Mascot (Perkins et al., 1999), MS-GF+ (Kim et al., 2010) and X!Tandem (Craig and Beavis, 2004) for each separate MS analysis. The corresponding FASTA containing protein sequences as well as a combination of all identification runs performed by PIA (Uszkoreit et al., 2019, 2015) and a peptide and protein quantification performed by OpenMS (Pfeuffer et al., 2017) is included. All data have been deposited to the ProteomeXchange Consortium via the PRIDE partner repository (Perez-Riverol et al., 2018) with the dataset identifier PXD012986.

## Specifications Table

**Table:** 

Subject	Omics: Proteomics
Specific subject area	Proteomics LC-MS/MS ground-truth dataset of quantitative spike-in proteins into complex matrix
Type of data	raw proteomics data identified spectra protein quantifications
How the data were acquired	Liquid chromatography coupled to tandem mass spectrometry (Q Exactive HF (Thermo Fisher Scientific) mass spectrometer operated in data dependent acquisition (DDA) mode performing HCD fragmentation)
Data format	Raw Analyzed
Description of data collection	C2C12 were grown in cell culture, harvested and lysed. The lysate was split into 5 aliquots. Each aliquot was spiked with 13 non-mouse proteins in varying amounts, keeping comparable overall sample amounts and physiologically plausible protein concentrations. The samples were measured in triplicates by LC-MS/MS in DDA mode and analyzed using peptide identification and quantification.
Data source location	Institution: Ruhr University Bochum, Medical Proteome Center (MPC) City: Bochum, NRW Country: Germany Latitude and longitude: 51.44539258N, 7.25739272 E
Data accessibility	Repository name: PRIDE [8] Data identification number: PXD012986 https://www.ebi.ac.uk/pride/archive/projects/PXD012986
Related research article	Barkovits K, Pacharra S, Pfeiffer K, Steinbach S, Eisenacher M, Marcus K, Uszkoreit J, Reproducibility, specificity and accuracy of relative quantification using spectral library-based data-independent acquisition. Mol Cell Proteomics. 2020 Jan;19(1):181–197. https://doi.org/10.1074/mcp.RA119.001714

**Value of the Data**

• This dataset contains a stable C2C12 cell line background and 13 spiked-in proteins in well annotated amounts analyzed by data dependent acquisition (DDA).
• In comparison to most other proteomics spike-in datasets, the proteins are added in physiological abundance to reflect realistic samples.
• The dataset can be used by scientists to evaluate workflows for the bioinformatics and statistics analysis of LC-MS/MS data and benchmark the results against well annotated ground truth data.
• This dataset can be considered as a reference for the development of new DDA analysis tools.

## 1 Data Description

The dataset described in this article contains a background of lysed and tryptically digested C2C12 (mouse) myoblast cells, into which 13 spike-in proteins (following referred to as ‘spikes’) were added in varying concentrations at five different states. The spikes were chosen from available proteins which originate from other species than mouse. They usually do not occur in the underlying C2C12 cells or their sequences overlap only to a very small amount. The set consists of six human proteins (α-synuclein, Fibrinogen α, β and γ as well as Hemoglobin α and β), three lipases (1,2, and 3) of *Candida rugosa*, bovine β-lactoglobulin, glucose oxidase of *Aspergillus niger*, chicken lysozyme C, and horse myoglobin. Some of the proteins were always spiked-in together (the fibrinogens, hemoglobins and lipases), as they derived from the same solutions. Besides having different molecular weights and protein sequence lengths, the proteins also were deliberately chosen to exhibit different grades of challenges during the MS analysis: not all of them produce tryptic peptides which could be ionized and measured by the mass spectrometer at all spike-in amounts, especially the lower concentrations where missed several times.

In total, five different spike-in combinations where generated and measured in triplicates. The total spike-in amount was kept as constant as possible between the different states while the protein concentrations of the spikes ranged from 0.1 to 10 pmol to reflect physiological states and have no influence on the measurement of the constant C2C12 background. In Table 1 the actual spike in proteins and their respective spike-in amounts for the five different states are given.

**Table 1:** Concentrations of the 13 spiked-in proteins per sample. Each protein (group) was spiked in the concentrations 0.1, 0.5, 1, 5 and 10 pmol in one sample, while the overall amount of spike-in proteins was kept as constant as possible.

		Amount of spike-in proteins (pmol)				
	UniProt Accession	Sample 1	Sample 2	Sample 3	Sample 4	Sample 5
**α-synuclein (pmol)**	P37840	1	10	0.5	0.1	5
**β-lactoglobulin (pmol)**	P02754	0.5	0.1	5	10	1
**Fibrinogen (pmol)** **α, β, γ each**	P02671, P02675, P02679	10	5	1	0.5	0.1
**Glucose oxidase (pmol)**	P13006	0.1	1	10	5	0.5
**Hemoglobin (pmol)** **α, β each**	P69905, P68871	0.5	5	10	1	0.1
**Lipase (pmol)** **1, 2, 3 each**	P20261, P32946, P32947	0.1	0.5	1	5	10
**Lysozyme (pmol)**	P00698	5	10	0.1	0.5	1
**Myoglobin (pmol)**	P68082	1	0.1	5	10	0.5

Besides the raw data from the mass spectrometer, the dataset already provides centroided mzML conversions (generated by msConvert [9]). Mapping of the raw files to the actual spike-in and replicate type is shown in Table 2, but also more thoroughly provided using SDRF [10] (compare also project page https://github.com/bigbio/proteomics-metadata-standard/tree/master/annotated-projects). For the spectrum identification a protein database as FASTA file is provided, which contains the UniProt [11] proteome for *mus musculus* (version 2017_12 containing 52,548 protein entries in total of which 16,946 are reviewed Swiss-Prot entries and 35,602 are derived by TrEMBL), together with the sequences of the spike-in proteins (also UniProt version 2017_12), the iRT proteins, the contaminants from cRAP (ftp.thegpm.org/fasta/cRAP/, version 2009-05-01) and several proteins identified as contaminants of the spike-in protein mixtures. Decoy entries were added, using shuffling of the original protein sequences.

**Table 2:** Mapping of the raw file name to the spike-in and replicate state.

	state 1	state 2	state 3	state 4	state 5
replicate 1	QExHF04026	QExHF04028	QExHF04030	QExHF04032	QExHF04034
replicate 2	QExHF04036	QExHF04038	QExHF04040	QExHF04032	QExHF04044
replicate 3	QExHF04046	QExHF04048	QExHF04050	QExHF04042	QExHF04054

Workflows for the analysis using KNIME are provided. These contain the spectrum identification using the search engines Mascot [2], MS-GF+ [3] and X!Tandem [4], for which the results in mzIdentML format are deposited as well. Furthermore, a quantification analysis is given on protein and peptide level, together with the results in CSV files.

## 2 Experimental Design, Materials and Methods

### 2.1 Sample Preparation

Frozen C2C12 cells were taken into culture in a 58 cm² petri dish (Sarstedt, Nümbrecht, Germany) within 10 ml DMEM (Gibco®, Thermo Fisher Scientific, Germany) standard medium containing 15% (v/v) FCS (Gibco®), 2% (v/v) sodium pyruvate (Biochrom, Berlin, Germany), 1% (v/v) non-essential amino acids (Biochrom) and 1% (v/v) penicillin/streptomycin (Pan Biotech, Aidenbach, Germany). The cells were cultivated at 37 °C and 5% CO2, medium was changed every two days and the cells were split at a confluency of approx. 70%. For the split, the cells were washed with 5 ml PBS (Gibco®), detached with 1.5 ml 0.05% Trypsin/1 M EDTA (Gibco®) for 3 min inside the incubator, finally the activity of trypsin was stopped by the addition of standard medium.

Cells were pelleted by centrifugation at 16,000x g for 10 min and then lysed in 30 mM TrisHCl, pH 8.5, 7 M urea and 2 M thiourea using glass beads and sonication (4 × 1 min on ice). After lysate, the sample was transferred into a fresh tube, glass beads were washed with distilled water, the resulting solution was combined with the lysate (resulting in 5.3 M urea and 1.5 M thiourea concentrations) and cleared by centrifugation at 16,000x *g* for 10 min.

To generate the different spike-in state samples, a constant amount of C2C12 lysate (20 µg) was spiked with varying amounts of the 13 spike-in proteins in 50 mM ammonium bicarbonate (AmBic) as specified in Table 1.

After reduction with dithiothreitol (DTT, final concentration of 5 mM) for 20 min at 56 °C, proteins were alkylated with iodoacetamide (13.75 mM final concentration) at ambient temperature for 30 min in the dark. Samples were diluted with 50 mM AmBic to an urea concentration < 1.5 M and digestion was carried out using trypsin (Serva, Heidelberg, Germany) at an enzyme to substrate ratio of approx. 1:27 at 37 °C overnight. The digestion was stopped by adding trifluoroacetic acid (TFA) to a final concentration of 0.5%. After centrifugation the supernatant was collected, and the peptide concentration was determined by amino acid analysis (AAA) as described previously [12]. For better comparison to other samples, the iRT kit provided by Biognosys (Schlieren, Switzerland) was added according to the manufacturer's instructions. In brief, solubilized iRT peptides were diluted 1:10 in 0.1% TFA and 1 µl was added to each sample.

To check the purity of the spike-in proteins, tryptic digestions of samples containing only the diluted proteins were analyzed on shorter LC-MS gradients (data not provided). The MS data was identified using reference proteome sets of the specific species (UniProt release 2017_03), which was used as expression host and/or the species from which the respective protein was expressed. For the identification of contaminants, an FDR of 1% using the target decoy approach was performed. For the generation of decoys, the original sequences were shuffled and the decoy database concatenated to the targets prior to spectrum identification. Altogether, 160 additional protein accessions were identified with valid peptide identifications. Some of these had high sequential overlap with the corresponding spike-in protein like respective isoforms, but several can best be explained as originating from unspecific purification.

### 2.2 Mass Spectrometry

For LC separation the nanoHPLC system Ultimate 3000 (Thermo Fisher Sceintific) was used with a PepMap 100 C18 (100 µm ID x 2 cm, particle size 5 µm, pore size 100 Å; Thermo Fisher Scientific) as precolumn and a PepMap C18 (75 µm x 50 cm, particle size 2 µm, pore size 100 Å; Thermo Fisher Scientific) as analytical column. Per sample 200 ng peptide amount as measured by the AAA was analyzed. Peptides were separated by a 120 min gradient using 0.1% formic acid (FA) as buffer A and 84% ACN in 0.1% FA as buffer B. The gradient was run from 5 to 40% buffer B. Subsequently, peptides were ionized by electrospray ionization and transferred into a Q Exactive HF mass spectrometer (Thermo Fisher Scientific). The capillary temperature was set to 250 °C and the spray voltage to 1600 V. The lock mass polydimethylcyclosiloxane (445.120 m/z) was used for internal recalibration.

The mass range of MS1 full scans was set to 350–1400 m/z with a resolution of 60,000 at 200 m/z (AGC 3 × 106, 80 ms maximum injection time). HCD fragmentation of the Top10 abundant precursor ions was performed at 27% NCE. The fragment analysis (MS2) was performed with a resolution of 30,000 at 200 m/z (AGC 1 × 106, 120 ms maximum injection time, 2.2 m/z isolation window).

### 2.3 Data Analysis

The resulting raw files were analyzed using workflows OpenMS [7] and PIA [5], [6] inside KNIME (workflows are provided). For this, the raw files were converted to mzML using msConvert and were searched by Mascot, MS-GF+ and X!Tandem using the following settings:

- As fixed modification, only carbamidomethylation at C was set, while as variable modifications oxidation (M), Gln->pyro-Glu (N-terminal Q), deamidated (NQ), ammonium (DE) and ammonia-loss (N, N-terminal C) were allowed due to sample preparation.
- A maximum of two missed cleavages was allowed.
- The precursor tolerance was set to 5 ppm and the fragment tolerance to 20 mmu.
- For cleavage Trypsin (cleavage at each K and R, unless followed by P) was used.
- The provided protein sequence database as FASTA was used.

The single searches per run were combined using PIA, after applying an FDR threshold of 1%. For the quantification, peptide features were detected using the FeatureFinderMultiplex and mapped to the identifications. Afterwards, alignment and normalization were performed by the appropriate OpenMS tools. Prior to the protein quantification using Top3 peptide abundancies, protein inference was conducted using PIA on all identification of all MS runs. The quantities for purely sequence based peptides ware inferred from the quantities of peptides distinguishing different modifications and charge states by summing up the respective raw quantities, which is the default approach in OpenMS. The resulting peptide and protein quantifications are provided as CSV files.

A statistical analysis on peptide and protein level was conducted. For this, all missing values were imputed to a value of 0 first. Afterwards, the data were transformed using the inverse hyperbolic sine function (arcsinh), which has similar characteristics as the logarithm in the given numeric range but is defined for 0. Afterwards, an analysis of variance (ANOVA) model was fitted to the transformed data. As a post-hoc test Tukey's honest significance test was conducted, to determine, which spike-in states were significantly differential. Finally, the ANOVA p-values were corrected for multiple testing using the Benjamini-Hochberg procedure. These results are also provided in CSV files for further analyzes.

While we will not give a detailed analysis of the quantified proteins in the dataset, we give a short overview in the following. In total, using the identification and quantification workflow as well as the statistical analysis described in [1], the dataset yields 3074 quantified protein groups. 2011 of these groups were quantified in each of the 15 MS analyzes with abundancies greater than 0 (respectively NA or null). From these groups, only the spiked-in proteins and any possible contaminants (see also above) should show any regulation, which due to measurement noise or processing artefacts (e.g., normalization) is not the case.

## Ethics Statements

For the sample preparation and analyzes described in this manuscript, cell culture models (C2C12 mouse cells) and purified commercially available spike-in proteins were used. No human or other animal material was used. Hence the manuscript adheres to the “Ethics in publishing” standards.

## CRediT authorship contribution statement

**Julian Uszkoreit:** Conceptualization, Methodology, Software, Writing – original draft, Data curation. **Katalin Barkovits:** Conceptualization, Methodology, Writing – original draft. **Sandra Pacharra:** Methodology, Conceptualization. **Kathy Pfeiffer:** Investigation. **Simone Steinbach:** Conceptualization. **Katrin Marcus:** Supervision, Funding acquisition, Resources, Writing – review & editing. **Martin Eisenacher:** Supervision, Funding acquisition, Project administration, Writing – review & editing.

## Declaration of Competing Interest

The authors declare that they have no known competing financial interests or personal relationships that could have appeared to influence the work reported in this paper.

## Data Availability

PXD012986: Reproducibility, specificity and accuracy of DIA quantification - DDA analysis https://www.ebi.ac.uk/pride/archive/projects/PXD012986.
